# Has the COVID-19 Pandemic Affected MPs’ Representational Activities?

**DOI:** 10.1017/S0008423920000566

**Published:** 2020-06-09

**Authors:** Royce Koop, Kelly Blidook, Lesley Anne Fuga

**Affiliations:** 1 Department of Political Studies, University of Manitoba, 532 Fletcher Argue, University of Manitoba, Winnipeg MB R3T 2N2; 2 Department of Political Science, Memorial University, Science Building Room 2028, Memorial University, St. John's NL A1B 3X9

## Abstract

The COVID-19 pandemic has necessarily affected the operation of Canada's Parliament and, thus, the activities of Members of Parliament (MPs) (Malloy, 2020; Rayment and VandenBeukel, 2020). Here, we explore how the pandemic has affected the representational activities of individual MPs.

The COVID-19 pandemic has necessarily affected the operation of Canada's Parliament and, thus, the activities of Members of Parliament (MPs) (Malloy, 2020; Rayment and VandenBeukel, 2020). Here, we explore how the pandemic has affected the representational activities of individual MPs.

In doing so, we rely on the Representational Connections Framework (RCF) developed by Koop, Bastedo and Blidook (2018) in their observational study of Canadian MPs. Like Fenno (2003), we conceive of representation as a series of connections that MPs develop and nurture between themselves and their constituents. The RCF shows how MPs tailor representational styles through their relative emphasis on four types of such connections: policy, service, symbolic and party connections (Koop et al., 2018: 19–23).^1^ MPs have significant autonomy and agency to develop their own representational styles, and they do so in response to several influences.

We view the COVID-19 pandemic as a new influence that could shape the representational behaviours of MPs. We conducted semistructured interviews with 11 MPs during May 2020. Questions were designed to probe MPs’ self-observed differences in their activities due to the COVID-19 pandemic, with an emphasis on connections with their constituents and how they typically spend their workdays at home and in Ottawa.^2^ The broad themes we present were highly consistent across interviews, although findings may not be generalizable due the relatively small sample.^3^

First, we discuss how the pandemic has shuffled MPs’ representational priorities, shifting MPs away from some connection-building activities and toward behaviours that support service connections. Second, we explore the particularities of service representation during the pandemic. The pandemic has affected MPs’ daily work through increased constituent demands for service in an abruptly changed operating environment. MPs report increased volume of constituent inquiries and requests, a changed nature in these demands as constituents mostly seek assistance and information related to COVID-19, and significant implications of a remote workstyle for MPs’ daily activities and those of their staff.

## COVID-19 Shifted MPs’ Representational Priorities toward Service

We found that COVID-19 has affected MPs’ connection-building representational activities. Most importantly, the pandemic resulted in an increased emphasis on constituents’ requests for service from MPs, which limited the diversity of representational practices normally found among MPs. While some MPs already prioritize service connections, this became the case during the pandemic for all MPs we interviewed, supplanting other types of connections. As MP Francis Scarpaleggia puts it: “We're in full service mode as opposed to, say, debate mode.”

While some MPs indicated a decline in their usual policy work, others said there has been no decline, but that increased service demands have simply increased the hours of work overall and/or altered what gets prioritized. “I think the amount of legislative work we're doing has not really diminished,” argues a government MP. “It's pretty much the same except we're also doing an influx of constituency work, a volume of constituency work that's increased quite dramatically.”

The physical distancing regulations and restrictions on public gathering necessitated by COVID-19 make it difficult for MPs to build and maintain symbolic ties with their constituents by attending events and being present in the community. As MP Ken McDonald notes,

“I miss the face-to-face because I could be at a senior's dinner in Carbonear and there could be 150 people there. So you get to mingle and speak to a lot of them face-to-face. Most of it is a cordial greeting, or someone might have an issue that they want to talk to you about. So this doesn't get done face-to-face now.”

“Politics is like many other human activities: it's a human-to-human interaction,” MP Kenny Chiu expands. “I need to have coffee with, I need to talk to, I need to shake hands … [with] constituents, stakeholders, [and] colleagues.” And MP Alexandra Mendès links the importance of face-to-face communication to characteristics of her constituency, arguing that “non-verbal communication is extremely important” for interacting with constituents in her highly diverse urban riding.

In response, many MPs have adopted new techniques to keep in touch and maintain linkages with constituents. MPs talked about webinar town halls and other online forums as ways to continue communications. MP Jenica Atwin, for example, reports that 160 people had attended a recent local webinar.

While some MPs are eager for the pandemic to end so they can get back to their normal operations, others noted that some of the shifts in their representational approaches may outlast the pandemic. MP Churence Rogers, for example, suggests

“… there's a lot of things about this COVID pandemic that will maybe change the way that I do the job in the future.” He expands, “… [T]o leave my home and drive to Grand Bank, it's like four hours. So if [mayors and municipal councillors] want a meeting about a particular issue, my executive assistant and I can set up a Zoom meeting…. And we can spend extra time in the meeting because now we don't have to spend eight hours driving.”

We also heard from some MPs that they are developing new representational connections with various local populations. MP Iqra Khalid, for example, reports that the pandemic has resulted in requests for assistance from local businesses, which generally have not contacted the MP's office in the past.

## Service Representation during the Pandemic

The predominant shift in representational behaviours reported by the MPs we interviewed is in their approach to building and maintaining service connections with their constituents. We observed three main ways the daily service work of MPs has changed.

First, MPs’ volume of casework increased dramatically. Regardless of whether they typically prioritize service connections, all the MPs we interviewed were experiencing surges in service needs, with many adding that their Ottawa staff are currently assigned to constituency matters. As MP Atwin explains, “Our offices have never been busier … phone calls, emails, or messages through the various platforms, so there's lots of points of contact every day … the volume has certainly increased exponentially.”

Second, the substance of the service work performed by MPs and their staff has changed during the pandemic. Typically, MPs and their staff develop the knowledge and skills to help constituents with a fairly narrow set of concerns. COVID-19 quickly spawned a host of government programs and regulations, generating new service concerns from constituents and increasing the immediacy with which offices need to respond.^4^ As an NDP MP notes,

My caseworkers have been inundated with casework. Normally, the majority of casework that comes is around immigration.… [N]ow, the vast majority, on top of the immigration pieces that we've been helping people with, is actually COVID-19-related questions. Either people who are trying to access the benefits that are available through the federal government or a lot of people who are falling through the cracks of those programs.

MP Mike Lake similarly adds that other service cases have often taken a backseat: “… [T]he priority from the government side is around COVID and so the typical things that we've dealt with … are completely different in terms of timeframes. Basically, a lot of the regular stuff is on hold.” The urgency of COVID-related service needs was emphasized by many of the MPs we interviewed. “This has to get done now,” said MP Raquel Dancho of these requests. “There is no putting this off. People are suffering, people are not being heard.”

Finally, COVID-19 has changed the way MPs gather and act on concerns. Normally, MPs receive service requests via email, phone calls, walk-ins to constituency offices, and unplanned or chance meetings at events or on the street. Staff or the MP then work with constituents to address these concerns. When COVID-19 resulted in the closing of riding offices, in addition to adapting to the increased volume and novelty of service requests, MPs and staff alike had to adjust.

For the most part, adjusting has meant working remotely from home. This entails increased use of technology, more staff and caucus meetings, and sometimes working without a dedicated office space among the demands of family. While MPs generally reported that operational practices transitioned well from constituency offices to remote settings, the loss of foot traffic deprived MPs of in-person contact with constituents, which is a key element of some members’ representational style.

## Conclusion

While MPs’ representational styles focus to varying degrees on policy, service, symbolic and party connections, the COVID-19 pandemic has tended to shift MPs’ behaviours heavily toward service. Across parties and jurisdictions, MPs emphasized the need to provide service to aid and inform constituents on matters related to COVID-19. In this way, COVID-19 has affected the nature of representational work undertaken by MPs during the crisis. The loss of opportunity to make significant strides in non-COVID-related policy and symbolic connection building during the “crisis” portion of the pandemic was discussed most by those MPs who prioritize those types of connections, and their frustration was often evident.

There is preliminary evidence that the changes we observed in Canada are also taking place in other democracies that employ single-member units of representation. In a larger study of representational practices currently underway, MPs in Australia and New Zealand told us that similar restrictions to those implemented in Canada caused a focusing of their time and energy toward direct service to constituents. Also similarly, while many saw benefits to take forward from their experiences with new media, most MPs expressed concern about a loss of in-person contact with constituents.

Once in office, MPs engage in experiential learning about being a representative or learning through reflection on doing (Koop et al., 2018: 24–25). Over time, such learning shapes MPs’ representational behaviours. The COVID-19 pandemic constitutes a dramatic period of learning for Canada's MPs. This study illustrates how MPs have been both flexible and responsive to the changes in representational demands posed by COVID-19. It also provides an opportunity to revisit the views of MPs from the first few months of the COVID-19 pandemic once it is clearer what its long-term effects upon politics and society will be.

## Notes

1 Policy connections are connections built through MPs listening to the policy concerns of constituents and communicating those to Parliament. Service connections are developed when MPs work to learn about problems that their constituents have and then assist constituents by addressing those problems. MPs often use the tools of constituency casework, notably staff focused on local issues working out of constituency offices, to develop service connections. Symbolic connections are psychological linkages developed through time spent in the riding, attending events and functions, being seen and developing personal relationships. Party connections refer to those built between the MP and party officials and colleagues both in the riding and in Ottawa.

2 We interviewed 11 of the 150 Canadian MPs invited. Interviews were conducted via phone/online by the authors. The sample includes MPs from four parties (Liberal, CPC, NDP, Green); from all regions except the North; women and men; from urban, rural and suburban ridings; and with a wide range of experience. Please contact the authors for more information on the sample and interview questions.

3 Notably, this research was motivated by data that began appearing in a larger study of representational practice and that align generally with these results. We accordingly are convinced we achieved saturation on the broad themes identified by our interviews and that these would not change significantly if we conducted further interviews.

4 The vast majority of service requests were for repatriation of constituents or family members who were stranded abroad, and questions pertaining to health and economic welfare concerns.
